# Seizure Type Classification Based on Hybrid Feature Engineering and Mutual Information Analysis Using Electroencephalogram

**DOI:** 10.3390/e27101057

**Published:** 2025-10-11

**Authors:** Yao Miao

**Affiliations:** College of Health Science and Environmental Engineering, Shenzhen Technology University, Shenzhen 518118, China; miao@sip.tuat.ac.jp

**Keywords:** seizure type, electroencephalogram (EEG), multi-band features, mutual information (MI), machine learning

## Abstract

Epilepsy has diverse seizure types that challenge diagnosis and treatment, requiring automated and accurate classification to improve patient outcomes. Traditional electroencephalogram (EEG)-based diagnosis relies on manual interpretation, which is subjective and inefficient, particularly for multi-class differentiation in imbalanced datasets. This study aims to develop a hybrid framework for automated multi-class seizure type classification using segment-wise EEG processing and multi-band feature engineering to enhance precision and address data challenges. EEG signals from the TUSZ dataset were segmented into 1-s windows with 0.5-s overlaps, followed by the extraction of multi-band features, including statistical measures, sample entropy, wavelet energies, Hurst exponent, and Hjorth parameters. The mutual information (MI) approach was employed to select the optimal features, and seven machine learning models (SVM, KNN, DT, RF, XGBoost, CatBoost, LightGBM) were evaluated via 10-fold stratified cross-validation with a class balancing strategy. The results showed the following: (1) XGBoost achieved the highest performance (accuracy: 0.8710, F1 score: 0.8721, AUC: 0.9797), with γ-band features dominating importance. (2) Confusion matrices indicated robust discrimination but noted overlaps in focal subtypes. This framework advances seizure type classification by integrating multi-band features and the MI method, which offers a scalable and interpretable tool for supporting clinical epilepsy diagnostics.

## 1. Introduction

Epilepsy is one of the most common chronic neurological disorders characterized by unpredictable recurrent seizures, affecting around 70 million people worldwide [1,2,3]. Seizure types vary widely, ranging from focal to generalized, such as focal non-specific seizure (FNSZ), absence seizure (ABSZ), complex partial seizure (CPSZ), tonic clonic seizure (TCSZ), generalized non-specific seizure (GNSZ), and tonic seizure (TNSZ) [4]. These various seizure types are with distinct pathophysiological mechanisms that necessitate precise classification for effective treatment, including antiepileptic drugs and surgical interventions [5,6]. Accurate classification of seizure types is essential for guiding appropriate treatment strategies [7]. Nonetheless, traditional diagnosis relies heavily on clinical expertise and visual EEG inspection, which is time-consuming, subjective, and prone to variability [8,9,10].

Electroencephalogram (EEG) serves as a primary non-invasive tool for capturing epileptic brain activity, offering high temporal resolution to detect abnormal discharges [11,12,13]. However, manual analysis of EEG signals is inefficient and error-prone. Existing methods often focus on binary classification, especially in seizure detection between seizure and non-seizure or single-band analysis [14,15,16,17,18]. A few studies focus on seizure type classification. One study focused on classifying three seizure types of GNSZ, FNSZ, and TCSZ, demonstrating the feasibility of automated classification by a support vector machine (SVM) [19]. Another work computed statistical features such as mean and kurtosis, using an SVM with quadratic kernels for four-class seizure classification at 95% accuracy [20]. Building upon this foundation, one survey highlighted the emerging role of artificial intelligence in automatic seizure type classification, emphasizing the potential of machine learning algorithms to set benchmarks in multi-class scenarios [21]. Feature extraction techniques have also been a focal point, such as the study that compared wavelet-based methods to improve multi-class seizure classification performance, measured through F1-scores [22]. Despite these advances, challenges persist in multi-class seizure classification, including data imbalance such as FNSZ dominating over TCSZ, artificially constructed features, feature redundancy in high-dimensional EEG data, and limited interpretability of models.

This study addresses these pressing challenges by introducing a novel feature-wise and segment-wise framework for multi-class seizure type classification using hybrid EEG feature engineering and mutual information (MI) analysis. Our approach extracts a 65-dimensional feature set from δ, θ, α, β, and γ bands via segment-wise sliding windowing, incorporating time and frequency domain metrics. MI-based selection refines features to an optimal 30-feature subset, reducing redundancy while highlighting γ-band discriminators critical for epileptiform activity. To mitigate class imbalance without oversampling artifacts, we employ weighted ensemble models, including XGBoost. Rigorous experiments on a comprehensive EEG dataset evaluated seven classifiers including accuracy, F1 score, precision, recall, and AUC, with cross-validation ensuring robustness. The results reveal that our XGBoost-integrated pipeline achieves an exceptional AUC of 0.9797, surpassing existing methods by offering interpretable insights through MI-driven feature visualizations. This advancement provides a transparent, efficient diagnostic tool that enhances accuracy in high-dimensional imbalanced datasets, marking a significant step forward in supporting epilepsy diagnosis and treatment.

## 2. Materials and Methods

**Figure 1:**
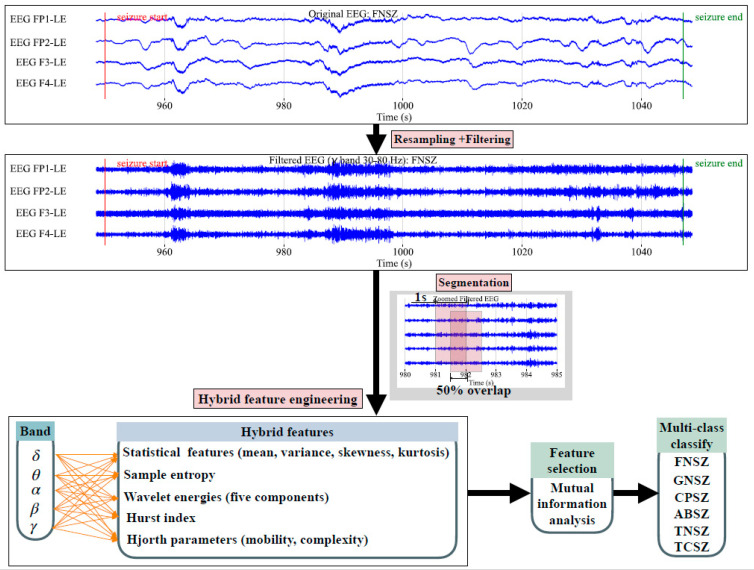
The proposed scheme. The upper section shows raw EEG signals of FNSZ with the x-axis as time (s) and y-axis as amplitude (μV), marking seizure start/end. The following is the resampling and filtering process. Then there is segmentation by dividing signals into 1 s segments with 50% overlap. After that is hybrid feature engineering by extracting statistical features (mean, variance, skewness, and kurtosis), sample entropy, wavelet energies, Hurst index, and Hjorth parameters from five frequency bands. Then there is MI analysis to select the top 30 features, leading to multi-class classification for ABSZ, CPSZ, FNSZ, GNSZ, TCSZ, and TNSZ.

As shown in Figure 1, the proposed framework for multi-class seizure classification processes EEG signals through resampling and bandpass filtering (0.5–100 Hz) to remove noise. Signals are then segmented into 1 s epochs with 50% overlap for enhanced temporal resolution. Hybrid feature engineering extracts multi-band features (δ, θ, α, β, and γ bands), including statistical measures, sample entropy, wavelet energies, Hurst exponents, and Hjorth parameters. Extracted features are followed by MI analysis to select the top 30 discriminative features to reduce dimensionality. Seven machine learning classifiers were evaluated using 10-fold stratified cross-validation, with classifiers incorporating a class weight strategy to address data imbalance, ensuring robust performance across all seizure types.

### 2.1. EEG Recordings and Preprocessing

This study employed the Temple University Hospital Seizure Corpus (TUSZ), a publicly available dataset containing labeled EEG recordings of epileptic seizures [23,24]. In this study, the EEG recordings of a total of 472 seizures for six seizure types were selected to be analyzed, which consisted of FNSZ, ABSZ, CPSZ, TCSZ, GNSZ, and TNSZ. The selection was guided by criteria aimed at achieving balanced representation and computational feasibility: (1) Prioritizing as many files as possible while capping at levels that prevented over-representation of abundant types. In our study, the selection included 100 files each for the more prevalent seizure types FNSZ, CPSZ, and GNSZ, while fully incorporating the less common types ABSZ (78 files), TCSZ (47 files), and TNSZ (47 files) to ensure comprehensive representation. (2) Ensuring multi-patient diversity to minimize biases. (3) Focusing on various durations sufficient for feature extraction, typically 14.66 s–1659.00 s, as summarized in Table 1. Each seizure EEG signal was segmented using a 1 s sliding window with a 0.5 s step size, increasing the sample size for robust analysis. The sampling frequency was resampled to 250 Hz, consistent with the dataset specifications. Detailed dataset statistics, including patient counts, seizure counts, total durations, and seizure-specific durations, are summarized in Table 1.

### 2.2. Multi-Domain Feature Extraction

Ictal EEG signals were decomposed into five sub-bands using the Finite Impulse Response (FIR) filter implemented in the MNE Python library (version 1.10.1, Alexandre, G., et al., Espoo, Finland), which ensures linear phase response and minimal distortion [25]. The five sub-bands consisted of δ (1–4 Hz), θ (4–7 Hz), α (8–12 Hz), β (13–30 Hz), and γ (30–80 Hz). For each band, a total of 13 features in the time domain, frequency domain, and non-linear domain were extracted, which included the features of mean, variance, skewness, kurtosis, sample entropy, five wavelet energies, Hurst Index, and Hjorth parameters (mobility and complexity). Specifically, the feature of mean for multi-band means was used to capture band-specific shifts, enhancing seizure discrimination. Then the filtered data were segmented using a 1 s sliding time window with 50% overlap. This resulted in a 65-dimensional-feature vector per segment. Features of all segments were standardized for subsequent analysis.

#### 2.2.1. Statistical Features

Four statistical features (mean, variance, skewness, and kurtosis) were utilized to evaluate EEG signal distribution in this study [26,27]. The mean was selected to calculate the central tendency of the EEG signal due to variations in mean values during seizures reflecting overall discharge intensity, aiding the differentiation of seizure types. The variance was used to measure EEG signal dispersion. Skewness and kurtosis assessed the asymmetry and peakedness of the signal distribution, respectively, aiding in identifying abnormal patterns and extreme values linked to seizure activity. The four statistical features were defined as

(1) $$\mu =\frac{1}{N}\sum_{i=1}^{N}x_{i},$$

(2) $$\sigma^{2}=\frac{1}{N}\sum_{i=1}^{N}{\left(x_{i}-\mu \right)}^{2},$$

(3) $$\gamma_{1}=\frac{\frac{1}{N}\sum_{i=1}^{N}{\left(x_{i}-\mu \right)}^{3}}{{\left(\frac{1}{N}\sum_{i=1}^{N}{\left(x_{i}-\mu \right)}^{2}\right)}^{3/2}},$$

(4) $$\gamma_{2}=\frac{\frac{1}{N}\sum_{i=1}^{N}{\left(x_{i}-\mu \right)}^{4}}{{\left(\frac{1}{N}\sum_{i=1}^{N}{\left(x_{i}-\mu \right)}^{2}\right)}^{2}}-3,$$

where *N* is the window signal length (250 samples), $x_{i}$ is the EEG data value, μ is the mean, $\sigma^{2}$ is the variance, and $\gamma_{1}$ and $\gamma_{2}$ represent skewness and kurtosis, respectively.

#### 2.2.2. Sample Entropy

Sample entropy is a non-linear feature to quantify the complexity of EEG data, which was selected as epileptic EEG signals are chaotic [28]. It can be defined as

(5) $$SampEn\left(m,r,N\right)=-\ln\left(\frac{A^{m}\left(r\right)}{B^{m}\left(r\right)}\right),$$

where *m* represents the embedding dimension, *r* is the tolerance threshold, $A^{m}\left(r\right)$ denotes the number of template matches for sequences of length *m*, and $B^{m}\left(r\right)$ represents the number of distance matches. In this study, we set m=2 and r=0.2∗σ, where σ is the standard deviation of the EEG signal. These values were selected based on established practices in epilepsy EEG analysis as they balance sensitivity to signal complexity and robustness to noise [29,30].

#### 2.2.3. Wavelet Energy

Wavelet energies are frequency-domain features from wavelet decomposition, which was selected to evaluate localized energy distribution [31]. Multi-band wavelet energies were computed to assess spectral differences. This could be computed via Daubechies-4 (db4) wavelet decomposition of the PyWavelets (version 1.8.0, Lee, G., et al.) library at four levels and was defined as [32]

(6) $$E_{j}=\sum_{k=0}^{L_{j}-1}c_{j,k}^{2},$$

where *j* is the decomposition level and $L_{j}$ is the length of the coefficient vector at level *j*.

#### 2.2.4. Hurst Index

Hurst index is a non-linear feature used to quantify the long-range dependence or self-similarity of EEG time series data [33]. It was selected in this study because epileptic EEG signals often exhibit persistent discharge patterns, where H>0.5 indicates positive long-term correlations. It can be defined as

(7) $$H=\frac{\log(R/S)}{\log(\tau )},$$

where R/S represents the rescaled range, representing the ratio of the range of the cumulative deviations of the EEG time series to its standard deviation over a given time interval. τ is the time scale, ranging from 2 to a maximum value $max_{lag}$. $max_{lag}$ was set to 20 to limit the maximum subinterval length to 20 samples in this study.

#### 2.2.5. Hjorth Parameters

Hjorth parameters are dynamic features to evaluate the mobility and complexity of an epileptic EEG waveform [34]. The formula is

(8) $$Mobility=\sqrt{\frac{Var(diff1)}{Var(x_{i})}},$$

(9) $$Complexity=\sqrt{\frac{Var(diff2)}{Var(diff1)}}/Mobility,$$

where diff1 and diff2 represent the first-order difference of two EEG signals. The Var(diff1) and Var(diff2) denote the variance of diff1 and diff2.

### 2.3. Feature Selection

To address the high dimensionality of the extracted EEG features, which included statistical measures (mean, variance, skewness, kurtosis), sample entropy, wavelet energies, Hurst exponent, and Hjorth parameters (mobility and complexity) across five frequency bands, a filter-based feature selection method utilizing MI was applied. MI quantifies the dependency between each feature *X* and the target class label *Y*, capturing both linear and non-linear relationships by measuring the reduction in uncertainty about *Y* given *X* [35]. For discrete variables, MI is formally defined as

(10) $$I\left(X;Y\right)=\sum_{x\in X}\sum_{y\in Y}p\left(x,y\right)\log\left(\frac{p(x,y)}{p(x)p(y)}\right),$$

where p(x,y) represents the joint probability mass function and p(x) and p(y) are the marginal probabilities. In the context of continuous EEG features, the mutual_info_classif estimator from scikit-learn was employed to compute non-parametric MI scores, approximating the joint and marginal distributions via k-nearest neighbors (KNNs) [36]. The SelectKBest algorithm then retained the top k=30 features with the highest MI scores, empirically chosen to balance model complexity, reduce overfitting, and preserve discriminative power while minimizing computational overhead. This threshold was determined through preliminary experiments, where k=30 yielded optimal cross-validation performance without significant information loss.

### 2.4. Classification Models

#### 2.4.1. Support Vector Machine (SVM)

A SVM is a supervised learning classifier, which constructs a hyperplane in a high-dimensional space to maximize the margin between classes while minimizing misclassification errors in a soft margin approach [37,38]. The objective function of a soft-margin SVM is defined as

(11) $$\min\frac{1}{2}{\left\|w\right\|}^{2}+C\sum \xi_{i},$$

where *w* is the normal vector, *C* is the regularization parameter, and $\xi_{i}$ are slack variables. In this study, the SVM was configured with a radial basis function (RBF) kernel to handle non-linear separability in multi-class seizure classification. The scikit-learn library was used and the class weight parameter was balanced to mitigate the imbalance inherent in the dataset for multi-class seizure types [36].

#### 2.4.2. K-Nearest Neighbor (KNN)

KNN is a lazy learning algorithm that classifies based on the majority vote of its *k* closest neighbors in the feature space, typically using Euclidean distance as the metric [39,40], defined as

(12) $$d\left(x,y\right)=\sqrt{\sum {\left(x_{i}-y_{i}\right)}^{2}},$$

where *d* is Euclidean distance. In this study, KNN was implemented with a neighbor number of 5, integrated with SMOTE for class balancing during cross-validation folds, following MI feature selection with k=30 to reduce dimensionality.

#### 2.4.3. Decision Tree (DT)

DT is a tree-based model that recursively splits data based on feature thresholds [41]. It was selected for its interpretability in exploring EEG feature hierarchies. It handles non-linear relationships well but is prone to overfitting, which can be mitigated through pruning or ensemble extensions. In our framework, a DT utilized a ‘balanced’ class weight parameter to address seizure type disparities.

#### 2.4.4. Random Forest (RF)

RF is an ensemble method that aggregates multiple decision trees trained on bootstrapped subsets with random feature sampling. It was chosen to reduce overfitting and improve robustness via majority voting [42]. It provides inherent feature importance and handles imbalance through class weighting. In this investigation, RF was parameterized with a balanced class weight parameter and evaluated after the selection of MI features. A balanced variant was also tested to further address class disparities. RF was chosen for its resilience to noise in high-dimensional EEG data, effectively modeling interactions among statistical, entropy, and wavelet features across bands.

#### 2.4.5. Light Gradient Boosting Machine (LightGBM)

LightGBM is an efficient gradient boosting implementation using histogram-based splitting and leaf-wise growth for reduced memory and faster training [43,44]. It incorporates GOSS and EFB for handling large datasets and imbalances. Configured with a ‘balanced’ class weight parameter, LightGBM utilized selected features and 10-fold validation. The classifier was selected for its scalability in high-dimensional EEG processing, prioritizing informative instances in band-specific features.

#### 2.4.6. Categorical Boosting (CatBoost)

CatBoost is a gradient boosting algorithm optimized for categorical features, employing ordered boosting and oblivious trees to reduce overfitting and bias [45]. It automatically handles class imbalance with ‘balanced’ weights. In this study, CatBoost was set with balanced auto class weights and integrated with MI selection (*k* = 30) and cross-validation. It was chosen for its efficiency in processing categorical-like EEG bands without encoding, effectively modeling complexities in entropy and mobility features.

#### 2.4.7. Extreme Gradient Boosting (XGBoost)

XGBoost is a scalable gradient boosting framework that sequentially builds trees to minimize a regularized loss function, incorporating techniques including shrinkage and column subsampling for enhanced generalization [46]. It supports multi-class objectives and handles imbalance via sample weights. Configured here with objective = ‘multi:softprob’ and inverse class frequency weights, XGBoost followed feature selection and 10-fold validation. The model was selected in this study for its superior performance in EEG analysis, capturing non-linear dependencies in features like Hjorth parameters and wavelet energies, and attributing to regularization that prevents overfitting in noisy, imbalanced datasets.

### 2.5. Evaluation Methodology

The performance of the proposed classifiers was rigorously evaluated using 10-fold stratified cross-validation, ensuring proportional representation of the six seizure classes (ABSZ, CPSZ, FNSZ, GNSZ, TCSZ, TNSZ) across folds to mitigate bias from dataset imbalance. For each fold, models were trained on nine subsets and tested on the held-out subset, with predictions aggregated for overall metrics. Evaluation metrics consisted of accuracy, weighted precision, weighted recall, weighted F1-score, and area under the receiver operating characteristic curve (AUC) [47,48].

Accuracy is an overall performance metric that evaluates the proportion of correct classifications. In this study, it was used to represent the model’s overall reliability in identifying seizure types. The accuracy was defined as

(13) $$Acc=\frac{TP+TN}{TP+TN+FP+FN}$$

The F1 score is the harmonic mean of precision and recall, used to balance imbalanced classes. In this study, it represented the model’s comprehensive performance on minority seizure types. The F1 score was defined as

(14) $$F1=2\times \frac{Precision\times Recall}{Precision+Recall}$$

Precision is the accuracy of positive predictions, evaluating the impact of false positives. In this study, it represented reducing misjudgments of seizures and avoiding unnecessary treatment. The formula was

(15) $$P=\frac{TP}{TP+FP}$$

Recall is the detection rate of true positives and evaluates omission risks. In this study, it represented reducing missed seizures and ensuring timely diagnosis. It was defined as

(16) $$R=\frac{TP}{TP+FN}$$

AUC is used to evaluate threshold robustness, where represents the overall ability to distinguish multi-class seizures. The AUC is defined as

(17) $$\mathrm{AUC}=\int_{0}^{1}TPR\left(FPR\right)dFPR$$

## 3. Results

### 3.1. Classification Results

The performance of seven machine learning models was evaluated using 10-fold stratified cross-validation on processed EEG segments, representing six seizure types: ABSZ, CPSZ, FNSZ, GNSZ, TCSZ, and TNSZ. Initially, features were extracted from five frequency bands (δ, θ, α, β, and γ), encompassing statistical measures (mean, variance, skewness, and kurtosis), sample entropy, wavelet energies, Hurst exponent, and Hjorth parameters (mobility and complexity). Subsequently, an MI-based feature selection method was applied to identify and retain the top 30 features, reducing dimensionality and enhancing generalization. Finally, these selected features were utilized for classification by the models. To establish a baseline for comparison, a null model was implemented, which randomly assigned seizure types with equal probability across the six classes, reflecting a naive approach with no feature-based learning. This model was evaluated using the same 10-fold cross-validation setup to provide a reference point for the performance of trained classifiers.

Table 2 presents the five performance metrics results (accuracy, weighted F1 score, precision, recall, and AUC), along with computational time for seven classifiers. Among the evaluated machine learning classifiers, XGBoost emerged as the top performer, achieving an accuracy of 0.8710 ± 0.0027, weighted F1 score of 0.8721 ± 0.0026, precision of 0.8744 ± 0.0025, recall of 0.8710 ± 0.0027, and AUC of 0.9797 ± 0.0007, with a computational time of 69.39s. The ensemble methods CatBoost followed as a close contender, recording an accuracy of 0.8641 ± 0.0034 and an AUC of 0.9789 ± 0.0009. RF also exhibited better classification performance, with an AUC of 0.9782 ± 0.0007. Methods with their efficiency enhanced by histogram-based optimization techniques, such as LightGBM, with an accuracy of 0.8595 ± 0.0030, also demonstrated strong performance. In contrast, simpler models, such as DT with an accuracy of 0.7899 ± 0.0054 and KNN with an accuracy of 0.5256 ± 0.0022, exhibited lower metrics. The SVM yielded the poorest results, with an accuracy of 0.4709 ± 0.0029. The null model, as expected, yielded an accuracy of 0.0149 and an AUC of 0.5000, consistent with random guessing for a six-class problem. This stark contrast highlights the effectiveness of the feature selection and classification approach.

To further validate the choice of sample entropy parameters used in feature extraction, we conducted a sensitivity analysis specifically on the top-performing XGBoost model. This analysis tested variations in m=1,2 and tolerance thresholds r=0.1,0.15,0.2∗σ to assess their impact on classification performance. The features of sample entropy were re-extracted using these parameter combinations, and XGBoost was retrained and evaluated under the same 10-fold cross-validation setup. The results, presented in Table 3, demonstrate that m=2 and r=0.2∗σ yielded the highest metrics across accuracy, F1-score, precision, recall, and AUC, confirming the optimality of these parameters for capturing epileptic EEG signal complexity.

Additionally, to determine the optimal number of features *k*, we evaluated the XGBoost model with k=10,20,40,50,60. As presented in Table 4, the results indicate that k=30 achieved a robust performance. While higher *k* values, such as k=60, yielded a slightly better AUC (0.9844 ± 0.0007), the improvement was marginal, with an increase of 0.0047, and the computational time rose from 69.39 s to 97.95 s. Performance improved steadily from k=10 to k=30, but, beyond k=30, the benefits diminished, suggesting potential noise or redundancy. The 10-fold cross-validation confirmed that k=30 provided an optimal balance between model effectiveness and computational efficiency.

Moreover, to further validate the feature extraction process, we investigated the impact of different step sizes on classification performance using the top-performing XGBoost model. Features were extracted with a fixed time window of 1 s and step sizes of 0.2, 0.4, 0.6, and 0.8 s, compared against the original step size of 0.5 s, all evaluated under 10-fold cross-validation. The results, presented in Table 5, confirm that the 0.5 s step size yielded the highest performance metrics (accuracy: 0.8701 ± 0.0027, AUC: 0.9797 ± 0.0007), outperforming the alternative configurations and justifying its selection.

### 3.2. Confusion Matrices

**Figure 2:**
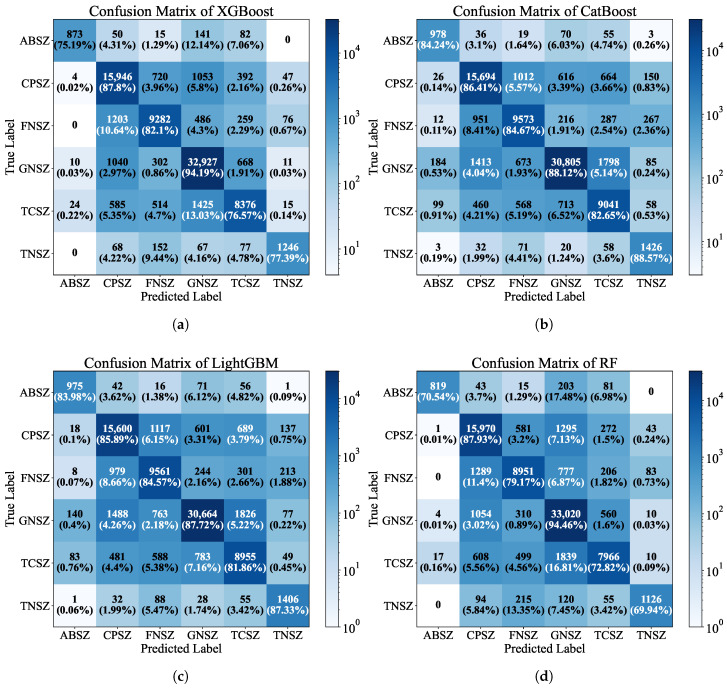
Confusion matrix for four classifiers, illustrating the classification performance across six seizure types (ABSZ, CPSZ, FNSZ, GNSZ, TCSZ, TNSZ). The x-axis represents the predicted labels and the y-axis represents the true labels, with colors indicating the log-scaled count of instances and numbers denoting the exact count of each segment classification outcome, accompanied by percentages representing the proportion of each count relative to the total per true label category. (**a**) Confusion matrix of XGBoost. (**b**) Confusion matrix of CatBoost. (**c**) Confusion matrix of LightGBM. (**d**) Confusion matrix of RF.

To enhance the understanding of model discriminability, confusion matrices were constructed for four classifiers with better performance (XGBoost, CatBoost, LightGBM, and RF), providing a detailed representation of true positives, false positives, and misclassifications of segments across the six seizure types. The matrices are presented in Figure 2. For the XGBoost model (Figure 2a), the high diagonal values indicated robust classification performance, with 9282 (82.1%) true positives for FNSZ and 32,927 (94.19%) true positives for GNSZ. However, a notable overlap was observed, with 720 (3.96%) CPSZ instances misclassified as FNSZ, suggesting shared electrophysiological characteristics within focal seizure subtypes. The CatBoost model (Figure 2b) achieved 9573 (84.67%) true positives for FNSZ and 30,805 (88.12%) for GNSZ, with 1012 (5.57%) CPSZ misclassified as FNSZ, further reinforcing the challenge of distinguishing focal seizures. The LightGBM model (Figure 2c) exhibited similar classification patterns, achieving 9561 (84.57%) true positives for FNSZ and 30,664 (87.72%) for GNSZ, but showed increased errors in rare classes, such as ABSZ, with 975 (83.98%) true positives and 42 (3.62%) misclassifications as CPSZ. This highlights the impact of class imbalance on rarer seizure types. In addition, the RF model (Figure 2d) recorded 8951 (79.17%) true positives for FNSZ and 33,020 (94.46%) for GNSZ, with 581 (3.2%) CPSZ instances misclassified as FNSZ, indicating a similar pattern of focal subtype confusion. Across all models, misclassifications predominantly occurred within focal subtypes, suggesting potential benefits from integrating hybrid features to improve separation. Zero values in the matrices, such as those between ABSZ and TNSZ in XGBoost, indicated no observed misclassifications, reflecting strong class-specific discrimination in certain cases.

### 3.3. Feature Importance Results

**Figure 3:**
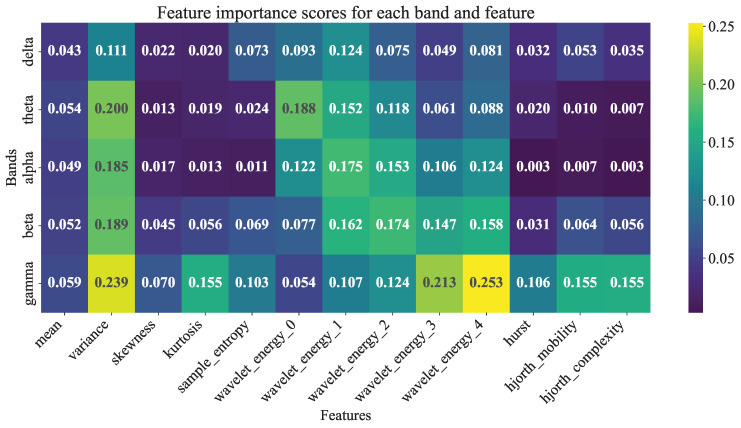
Feature importance scores heatmap of various EEG features across five frequency bands assessed by MI selection. The x-axis represents the feature types, including mean, variance, skewness, kurtosis, sample entropy, wavelet energy 0 through wavelet energy 4, Hurst index, Hjorth mobility, and Hjorth complexity. The y-axis denotes the five frequency bands (δ, θ, α, β, and γ). The color intensity, ranging from light to dark within the viridis color map, indicates the magnitude of MI scores, with darker shades representing higher scores (ranging from 0.003 to 0.253). Numbers within each cell, displayed in bold font, represent the specific MI scores, with higher values signifying greater feature importance in classifying seizure types, while zeros indicate no significant contribution.

The feature importance analysis was conducted by the MI method, providing insight into the discriminative power of EEG features across five frequency bands, as depicted in Figure 3. This heatmap visualizes feature importance scores within a 5 × 13 matrix for five frequency bands and 13 feature types, which include mean, variance, skewness, kurtosis, sample entropy, wavelet energy 0 through wavelet energy 4, Hurst exponent, Hjorth mobility, and Hjorth complexity. The results reveal that features in the γ band dominated the top rankings, with wavelet energy 4 at the γ exhibiting the highest feature importance score of 0.253, followed by variance at the γ band (0.239) and wavelet energy 3 at the γ band (0.213). This suggests that high-frequency oscillatory components, particularly wavelet-based energy measures, are critical for distinguishing seizure types in the EEG dataset. Other notable contributors include variance at the θ band (0.200) and variance (0.189) at the β band, indicating that variance across mid-frequency bands also plays a significant role. Within the γ band, Hjorth complexity (0.155) and mobility (0.155) further underscored the importance of non-linear dynamics in seizure characterization. Conversely, the α and δ bands showed lower feature importance scores, with Hjorth complexity at the α band (0.003) and Hurst at the δ band (0.032) ranking among the least influential, suggesting limited discriminative power in these ranges.

Common top features (e.g., gamma_hjorth_complexity, beta_hjorth_mobility) across ensembles suggested band-specific complexity metrics as robust discriminators, aligning with prior TUSZ studies where gamma features improved multi-class accuracy by emphasizing oscillatory irregularities. This analysis underscores the value of hybrid statistical–nonlinear features for epilepsy classification, potentially guiding feature engineering in future scalable systems.

## 4. Discussion

This study proposes a comprehensive framework for multi-class seizure classification, focusing on EEG segments from six seizure types (ABSZ, CPSZ, FNSZ, GNSZ, TCSZ, and TNSZ). Multi-band features (δ, θ, α, β, and γ) were extracted through segment-wise processing (1 s windows with 0.5 s overlap), encompassing statistical measures (mean, variance, skewness, kurtosis), sample entropy, wavelet energies, Hurst exponents, and Hjorth parameters. Feature-wise analysis was enhanced via MI-based selection to retain the top 30 features, while imbalance handling was addressed through class-weighted sampling. Evaluated via 10-fold stratified cross-validation, XGBoost emerged as the top performer, with an accuracy of 0.8710 ± 0.0027, F1-score of 0.8721 ± 0.0026, and AUC of 0.9797 ± 0.0007.

The classification results demonstrate robust performance in distinguishing six seizure types (ABSZ, CPSZ, FNSZ, GNSZ, TCSZ, TNSZ), particularly with gradient boosting models such as XGBoost and LightGBM, which outperformed baselines in handling imbalanced distributions. Confusion matrices revealed high true positives along diagonals, such as 32,927 for GNSZ in XGBoost, indicating strong generalization for prevalent classes. However, the 1203 CPSZ segments misclassified as FNSZ by XGBoost revealed subtle but discernible overlaps between distinct focal subtypes, suggesting hidden electrophysiological commonalities. Such overlaps may have been due to shared spike-wave patterns in temporal and frontal lobes [49].

Clinically, this implies the potential for precise antiepileptic therapy tailoring, reducing recurrence risks in idiopathic focal epilepsy by enabling early subtype-specific interventions. Conducting feature importance analysis by introducing the MI selection approach highlighted γ-band dominance, with wavelet energy in the γ band scoring 0.253, which underscores high-frequency energy fluctuations as key discriminators of seizure type [50,51]. Multi-band variance and wavelet features in the θ and β bands further contributed to capturing non-stationary signal variations that aligned with epileptiform oscillations for different seizure types.

There are four key limitations included in this study. Firstly, the dataset-specific biases, such as truncation artifacts, potentially inflated performance for common classes. Secondly, the offline nature restricted real-time deployment. Thirdly, feature selection, while effective, may have overlooked rare inter-band interactions. Fourthly, the current analysis relied on preprocessed EEG data with standard artifact rejection techniques such as bandpass filtering, but lacked specific robustness tests against simulated or real-world noise sources such as eye blinks, muscle activity, and electrode drift. This limits the assurance of model reliability in practical, noisy environments. Future work should incorporate larger datasets or raw EEG for improved generalization, focus on real-time analysis, and include comprehensive noise robustness testing with simulated artifacts and real-world scenarios to enhance the model’s applicability across diverse conditions. Furthermore, to maximize XGBoost’s strength in managing imbalanced classes, we plan to develop and test new statistical features designed to better represent the complex patterns in EEG data, particularly for rare seizure types in future work. Preliminary explorations indicate the potential for improved classification performance, and this integrated approach will strengthen the overall robustness and applicability of our method.

The proposed framework represents a significant advancement in epilepsy classification by combining multi-band feature extraction with MI-based selection, tailored to the imbalanced nature of seizure types. This integration, particularly the emphasis on γ-band features, distinguishes our approach from traditional methods that often focus on narrower frequency ranges or simpler feature sets [8]. The superior performance of XGBoost (accuracy: 0.8701 ± 0.0027, AUC: 0.9797 ± 0.0007) underscores the potential of this method to address clinical challenges. However, the clinical need for automated diagnostic tools remains pressing due to the labor-intensive nature of manual EEG interpretation and the risk of missed diagnoses. This highlights the importance of developing robust, automated systems to support neurologists.

In summary, this study developed a feature-wise and segment-wise framework for EEG analysis, leveraging multi-band feature extraction and an MI selection method, optimizing performance in high-dimensional, imbalanced data. This pipeline integrates segment-wise windowing for enhanced temporal resolution with band-specific features to detect frequency-dependent epileptiform traits, addressing class imbalance through weighted ensemble models like XGBoost, which achieved the highest AUC of 0.9797, without relying on oversampling artifacts, which often compromise generalization. Unlike traditional single-band or unsegmented methods, our methodology advances seizure classification by prioritizing interpretability through MI-based feature importance visualizations, highlighting γ-band features as key discriminators. This interpretability facilitates clinical adoption and diagnostic transparency, while the novel 65-dimensional multi-band EEG extraction pipeline, optimized to 30 features, effectively mitigates feature redundancy and imbalance. The comprehensive evaluation of seven models with tailored imbalance optimizations sets a new performance benchmark and provides a robust, automated diagnostic framework that reduces clinical workload and enhances accuracy, particularly in resource-limited settings.

## 5. Conclusions

This study proposed a novel framework for seizure type classification by integrating multi-band and segment-wise feature extraction with MI-based selection and strategies for handling imbalanced EEG data. Features were extracted across five frequency bands and encompassed statistical measures, sample entropy, wavelet energies, Hurst exponent, and Hjorth parameters. Moreover, the MI approach was employed to identify the most discriminative features for enhanced performance. Seven classifiers underwent rigorous evaluation through 10-fold stratified cross-validation. The results showed that XGBoost exhibited superior classification performance and the critical role of γ-band features in detecting epileptiform activity. Comprehensive assessments further affirmed the framework’s robustness in managing imbalanced datasets, strengthening classification outcomes. This approach provides a scalable and interpretable methodology, offering substantial advancements for clinical epilepsy diagnosis and supporting the development of tailored treatment strategies.

## Figures and Tables

**Table 1:** Summary of selected dataset information.

Seizure Type	Patients	Seizures	Total Duration(s)	Seizure Duration Range(s)
FNSZ	15	100	5726.88	15.95–1552.74
ABSZ	10	78	637.70	14.66–202.68
CPSZ	19	100	34,771.30	45.74–1478.29
TCSZ	6	47	839.45	40.62–748.94
GNSZ	20	100	59,616.00	21.35–1659.00
TNSZ	8	47	5502.97	49.90–748.94

**Table 2:** Seizure type classification performance metrics for the seven classifiers and the null model (values in bold indicate the highest values).

Classifier	Accuracy	F1-Score	Precision	Recall	AUC	Time (s)
SVM	0.4709 ± 0.0029	0.4618 ± 0.0034	0.6249 ± 0.0037	0.4709 ± 0.0029	0.8148 ± 0.0026	32,984.36
KNN	0.5256 ± 0.0022	0.5397 ± 0.0024	0.5870 ± 0.0038	0.5256 ± 0.0022	0.7665 ± 0.0031	65.80
Decision Tree	0.7899 ± 0.0054	0.7897 ± 0.0053	0.7896 ± 0.0053	0.7899 ± 0.0054	0.8544 ± 0.0039	102.09
LightGBM	0.8595 ± 0.0030	0.8612 ± 0.0029	0.8651 ± 0.0027	0.8595 ± 0.0030	0.9772 ± 0.0008	6208.87
RF	0.8684 ± 0.0022	0.8664 ± 0.0022	0.8686 ± 0.0023	0.8684 ± 0.0022	0.9782 ± 0.0007	228.99
CatBoost	0.8641 ± 0.0034	0.8658 ± 0.0033	0.8697 ± 0.0030	0.8641 ± 0.0034	0.9789 ± 0.0009	532.23
XGBoost	**0.8710 ± 0.0027**	**0.8721 ± 0.0026**	**0.8744 ± 0.0025**	**0.8710 ± 0.0027**	**0.9797 ± 0.0007**	69.39
Null model	0.0149 ± 0.0012	0.0004 ± 0.0001	0.0002 ± 0.0000	0.0149 ± 0.0012	0.5000 ± 0.0000	1.79

**Table 3:** Sensitivity analysis of sample entropy parameters (*m* and *r*) on XGBoost performance metrics (values in bold indicate the highest values).

*m*	*r*	Accuracy	F1-Score	Precision	Recall	AUC
1	0.1∗σ	0.8659 ± 0.0024	0.8672 ± 0.0024	0.8699 ± 0.0024	0.8659 ± 0.0024	0.9782 ± 0.0009
	0.15∗σ	0.8659 ± 0.0024	0.8672 ± 0.0024	0.8699 ± 0.0024	0.8659 ± 0.0024	0.9782 ± 0.0009
	0.2∗σ	0.8659 ± 0.0024	0.8672 ± 0.0024	0.8699 ± 0.0024	0.8659 ± 0.0024	0.9782 ± 0.0009
2	0.1∗σ	0.8693 ± 0.0035	0.8705 ± 0.0034	0.8729 ± 0.0033	0.8693 ± 0.0035	0.9793 ± 0.0008
	0.15∗σ	0.8699 ± 0.0036	0.8710 ± 0.0035	0.8733 ± 0.0035	0.8699 ± 0.0036	0.9796 ± 0.0007
	0.2∗σ	**0.8710 ± 0.0027**	**0.8721 ± 0.0026**	**0.8744 ± 0.0025**	**0.8710 ± 0.0027**	**0.9797 ± 0.0007**

**Table 4:** Performance metrics of XGBoost with varying numbers of top features (*k*).

*k*	Accuracy	F1-Score	Precision	Recall	AUC	Time (s)
10	0.7561 ± 0.0040	0.7602 ± 0.0037	0.7719 ± 0.0031	0.7561 ± 0.0040	0.9397 ± 0.0017	46.43
20	0.8463 ± 0.0030	0.8480 ± 0.0029	0.8521 ± 0.0029	0.8463 ± 0.0030	0.9729 ± 0.0009	53.16
30	0.8710 ± 0.0027	0.8721 ± 0.0026	0.8744 ± 0.0025	0.8710 ± 0.0027	0.9797 ± 0.0007	69.39
40	0.8868 ± 0.0027	0.8875 ± 0.0027	0.8889 ± 0.0027	0.8868 ± 0.0027	0.9836 ± 0.0007	77.17
50	0.8862 ± 0.0025	0.8869 ± 0.0025	0.8883 ± 0.0025	0.8862 ± 0.0025	0.9837 ± 0.0007	88.24
60	0.8899 ± 0.0027	0.8905 ± 0.0027	0.8917 ± 0.0026	0.8899 ± 0.0027	0.9844 ± 0.0007	97.95

**Table 5:** Performance metrics of XGBoost with varying step sizes (time window = 1 s, values in bold indicate the highest values).

Step Size (s)	Accuracy	F1-Score	Precision	Recall	AUC	Time (s)
0.2	0.8645 ± 0.0017	0.8662 ± 0.0016	0.8701 ± 0.0015	0.8645 ± 0.0017	0.9795 ± 0.0004	159.66
0.4	0.8583 ± 0.0020	0.8599 ± 0.0020	0.8631 ± 0.0021	0.8583 ± 0.0020	0.9766 ± 0.0010	80.60
0.5	**0.8710 ± 0.0027**	**0.8721 ± 0.0026**	**0.8744 ± 0.0025**	**0.8710 ± 0.0027**	**0.9797 ± 0.0007**	69.39
0.6	0.8532 ± 0.0057	0.8546 ± 0.0056	0.8572 ± 0.0054	0.8532 ± 0.0057	0.9741 ± 0.0015	57.22
0.8	0.8495 ± 0.0061	0.8507 ± 0.0060	0.8532 ± 0.0057	0.8495 ± 0.0061	0.9724 ± 0.0014	46.50

## Data Availability

The datasets analyzed for this study can be found in the TUSZ dataset by visiting the following link: https://isip.piconepress.com/projects/tuh_eeg/ (accessed on 28 September 2024).

## Abbreviations

The following abbreviations are used in this manuscript:

EEG	Electroencephalogram
FNSZ	Focal non-specific seizure
ABSZ	Absence seizure
CPSZ	Complex partial seizure
TCSZ	Tonic clonic seizure
GNSZ	Generalized non-specific seizure
TNSZ	Tonic seizure
MI	Mutual information
TUSZ	Temple University Hospital Seizure Corpus
SVM	Support vector machine
KNN	K-nearest neighbor
DT	Decision tree
RF	Random forest
LightGBM	Light gradient boosting machine
CatBoost	Categorical boosting
XGBoost	Extreme gradient boosting
AUC	Area under the receiver operating characteristic curve
